# The Use of Facebook Advertising for Communicating Public Health Messages: A Campaign Against Drinking During Pregnancy in New Zealand

**DOI:** 10.2196/publichealth.7032

**Published:** 2017-08-10

**Authors:** Mathew Parackal, Sherly Parackal, Shobhit Eusebius, Damien Mather

**Affiliations:** ^1^ Department of Marketing University of Otago Dunedin New Zealand; ^2^ Department of Preventive and Social Medicine University of Otago Dunedin New Zealand

**Keywords:** social media, health promotion, alcohol, pregnancy, New Zealand

## Abstract

**Background:**

Social media is gaining recognition as a platform for delivering public health messages. One area attracting attention from public health researchers and professionals is Facebook’s advertising channel. This channel is reported to have a broad reach and generate high user engagement with the disseminated campaign materials. However, to date, no study has examined the communication process via this channel which this study aimed to address.

**Objective:**

The specific objectives of the study were to (1) examine user engagement for a public health campaign based on the metadata provided by Facebook, (2) analyze comments generated by the campaign materials using text mining, and (3) investigate the relationship between the themes identified in the comments and the message and the sentiments prevalent in the themes that exhibited significant relationships.

**Methods:**

This study examined a New Zealand public health pilot campaign called “Don’t Know? Don’t Drink,” which warned against drinking alcohol during pregnancy. The campaign conveyed the warning through a video and three banner ads that were delivered as news feeds to women aged 18-30 years. Thematic analysis using text mining performed on the comments (n=819) identified four themes. Logistic regression was used to identify meaning-making themes that exhibited association with the message.

**Results:**

The users’ engagement was impressive with the video receiving 203,754 views. The combined likes and shares for the promotional materials (video and banner ads) amounted to 6125 and 300, respectively. The logistic regression analysis showed two meaning-making themes, namely, risk of pregnancy (*P*=.003) and alcohol and culture (*P*<.001) exhibited association with the message. The sentiment analysis carried out on the two themes revealed there were more negative than positive comments (47% vs 28%).

**Conclusions:**

The user engagement observed in this study was consistent with previous research. The numbers reported for views, likes, and shares may be seen as unique interactions over the fixed period of the campaign; however, survey research would be required to find out the true evaluative worth of these metadata. A close examination of the comments, employing text mining, revealed that the message was not accepted by a majority of the target segment. Self-identity and conformity theories may help to explain these observed reactions, albeit warrant further investigations. Although the comments were predominantly negative, they provide opportunities to engage back with the women. The one-way communication format followed in this campaign did not support any two-way engagement. Further investigation is warranted to establish whether using a two-way communication format would have improved the acceptability of such public health messages delivered via social media. The findings of this study caution using a one-way communication format to convey public health messages via Facebook’s advertising channel.

## Introduction

### Background

Social media websites accumulate a vast amount of user-generated content [1,2]. Of interest to public health researchers is health information placed on social media by individuals with health conditions and their families [3]. For example, people with diabetes voluntarily share diabetes management strategies on Facebook for the benefit of others enduring that condition [4]. Likewise, the narratives of cancer patients on YouTube offer valuable health messages to current and future patients [5]. Farmer et al [6] identified 757 dedicated health groups on Facebook comprising 290,962 individuals. The content generated by these health groups is readily available to individuals seeking health information. These networked systems allow participants to add value, correct misconceptions, and generate credible information within any given conversation [7]. Thus, social media platforms (Facebook in particular) are recognized as valuable sources for information on health topics [8-11].

### Use of Social Media in Public Health

The use of social media as an information source for health topics has opened channels of communication between health care providers and their patients. Both individual health care providers [12] and institutions [13,14] use social media to communicate with their clients. The notion that social media could operate like mass media [15] is also drawing the interest of public health researchers [16]. Whether social media can effectively communicate messages to better facilitate public health interventions requires investigation [17,18]. To date, literature on the effectiveness of social media to communicate public health messages is sparse but emerging, as evidenced by recent publications [19]. Early indications from randomized controlled trials (RTCs) [20-23] investigating social media’s effectiveness at communicating public health messages show promising results. One study [22] showed communication via Facebook to effectively sustain modified alcohol consumption behavior for up to 3 months, postintervention.

### Use of Facebook Advertising for Delivering Public Messages

Although support for using social media to communicate public health messages is gaining momentum, there are still areas that warrant further investigation. One such area is social media’s advertising channel. Social media sites are set up with the right to communicate with their users. Owners of these websites lease out this right to contact users within their system. Individuals and organizations lease out the right to disseminate messages, as news feed, to the accounts of their target audience. Researchers have successfully tested Facebook’s advertising channel for recruiting specific subjects to participate in health studies [24] (eg, women who are 8-10 weeks pregnant). This study [24] found Facebook’s advertising channel to be cost-effective and efficient for recruitment. The channel is valuable for communicating public health messages because of its broad reach. For example, a campaign to raise awareness for a newborn screening and bio-banking program used Facebook’s advertising channel to reach 1.88 million users from the US state of Michigan [25]. The campaign generated 9186 likes, 452 shares, and 642 comments. Whereas the engagement reported in Platt et al [25] was impressive, what remains unknown is how much of that translated into acceptance of the program.

This paper reports the findings of a study that examined the use of Facebook’s advertising channel as a vehicle for health promotion. The study was on a New Zealand public health campaign called “Don’t Know? Don’t Drink” [26], which is part of the New Zealand Health Promotion Agency’s (HPA’s) wider alcohol harm reduction program that aims to reduce the prevalence of alcohol-exposed pregnancies. This campaign aimed to convey the following message: “A woman who thinks there is a chance she may be pregnant should stop drinking alcohol until she knows she is not pregnant.”

The campaign targeted women aged 18-30 years, based on evidence that these women are at risk for drinking during pregnancy [27]. A video and three banner advertisements, carrying the message, were piloted from June to September 2015 using Facebook’s advertising channel.

The aim of this study was to better understand the communication process of Facebook’s advertising channel for conveying public health messages. The specific objectives of the study were to (1) examine user engagement for the video and banner ads based on metadata provided by Facebook, (2) analyze comments generated by the campaign materials using text mining, and (3) investigate the relationship between the themes identified in the comments and the message and the sentiments prevalent in the themes that exhibited significant relationships.

## Methods

### Metadata

The metadata provided by Facebook are designated as counts of likes, shares, and views. Likes and shares are provided for all postings, whereas views are provided for videos only. By clicking the like button, Facebook users convey their approval of a posting. Facebook displays the total number of likes received for a posting. The share button allows users to share a posting with others within their network. The total number of shares for a posting is also displayed. Views indicate the number of times a video was run. Although it is hard to assign specific evaluative worth, metadata does help more generally to assess the audience’s level of engagement with a campaign.

### Thematic Analysis

The comments made on postings contain textual information that, when classified and analyzed, indicate how and to what extent the target audience made meaning of and reflected the campaign’s embedded message. This study used text mining to reveal the meaning-making and message reflecting themes within the comments. As all the promotional materials were designed to convey a single message, the comments received for all the postings (both the video and banner ads) were combined and analyzed. The number of comments (n=819) was sufficient for conducting a thematic analysis. The methodology employed for the thematic analysis comprised first defining the corpus of words and then carrying out text parsing, stemming, filtering, and dimensional reduction via unsupervised and supervised classification of terms into topics or themes for meaning-making and message reflection, respectively.

#### Definition of the Corpus of Words

The extraction of comments from Facebook required expanding the conversation threads to make all the comments visible. Following this, an exact copy of the comments was captured by maintaining the format of the Facebook pages. The raw data was scrubbed down to remove all meta-information attached to the comments (eg, time stamps and likes) and redundant formats (eg, extra carriage returns, horizontal tabs, and empty rows). At the end of this scrubbing, the Facebook usernames and their comments were left in separate rows. Each row was converted into a text file containing only the name and the comment. There were as many text files as there were comments, forming the corpus of terms analyzed. The text files were imported into SAS Enterprise Miner 12.3 (SAS Institute Inc) for thematic analysis. The identifiable names attached to the comments were replaced by codes to ensure that the confidentiality of the data was maintained throughout the analyzing and reporting process so as to minimize any unintentional harm to the participants.

#### Text Mining of the Themes

The aim of the text mining was to discover underlying themes in the corpus of words. The text mining steps included text parsing, text filtering, and theme generation [28]. Text parsing broke down the comments into root words (or stemming), parts-of-speech, and term definition. By stemming, words containing the same root word were made into that root word (eg, “drink” and “drinking” were stemmed to “drink”). By defining the parts-of-speech, the role of each word in a sentence was identified, and, by term identification, a term-by-frequency matrix was created for the corpus of terms. The terms were assigned information weights using the inverse document frequency (IDF) method [29]. IDF assigns high weights (implying greater importance) to words that appear less frequently and low weights to those that appear more often (implying lesser importance). Text filtering reduced the number of terms to a more manageable number while minimizing the loss of information. This step removed words that carried little or no meaning according to their information weight while maintaining a minimum document incidence threshold of four. Themes were generated using the data reduction technique of singular value decomposition (SVD) [28] to identify and quantify the meaning-making that took place. SVD reduced the corpus into orthoginal dimensions to reveal the underlying themes in the data. The terms in the themes were assigned a “theme-term-weight.” The cut-off for inclusion in the theme was computed using the formula: “mean theme-term-weight plus one standard deviation” [28]. The terms with weights greater than the cut-off value formed the “keywords,” and they conveyed an approximated essence of the theme. Likewise, comments within a theme were assigned a “theme-comment-weight.” The cut-off point for inclusion of comments was calculated using the formula: “mean theme-comment-weight plus one standard deviation” [28]. The comments that received the highest weights formed the archetypes of that theme. By reviewing the keywords and the corresponding theme archetype comments, meaningful labels were then assigned to the themes.

The presence of the reflective message in the themes was identified using a supervised classification via a user-supplied topic-term-role based on the terms and grammar roles derived from the sponsoring stakeholder’s message. Each comment document was assigned a “message reflection theme information weight” using this method. The interval scale message measurement variable for the message reflection theme was then modified to produce a binary value based on the message reflection topic weight threshold, where “one” represented a strong evidence of the theme being present in the comments and “zero” represented otherwise. The dependent variable for this analysis was thus derived using the keywords of the message (see Textbox 1).

The information weight threshold and binary coding modifications explained above were applied to each of the comments to produce the dependent message reflection theme variable and the independent or predictor meaning-making theme variables. The critical alpha for inferential statistics for the predictive model used was .05, for the evidence of an association to be deemed to be strongly apparent.

**Keywords of the message.**

Message

- “A woman who thinks there is a chance she may be pregnant should stop drinking alcohol until she knows she is not pregnant.”

Keywords

- woman, think, chance, pregnant, stop, drink, know, not

### Logistic Regression

On social media, users express their ideas voluntarily. and the bias of social desirability has a surprisingly minimal influence. Such free-flowing comments convey what is on the users’ minds more honestly [1]. Using logistic regression, an investigation was carried out to find out whether the contents of the message were related to, or predicted by the comments. A stepwise method of predictor variable selection in the logistic regression model framework was used with a critical “stay” alpha value of .05. This value helped to identify the themes extracted from the comments in the unsupervised classification text mining phase which strongly exhibited associations with the desired supervised classification message theme. The approach, which comprises of identifying themes in the comments and using them in a logistic regression model to investigate for predictive associations, is gaining increasing recognition (eg, [30]). This study used logistic regression in the context of dependent and independent variables dervived from the unsupervised and supervised classification of the text mining phase.

### Sentiment Analysis

Sentiment analysis was used to explain the outcome of the logistic regression. The themes meeting the logistic regression threshold for strong evidence for the presence of the reflective message retained in the stepwise selection process were subjected to sentiment analysis using Mathematica 10.3 (Wolfram Research, Inc., Mathematica, version 10.3) [31]. The comments of the themes retained that met the message evidence threshold were categorized as positive, negative, and neutral. The authors reviewed the comments to verify the categorization was accurate, particularly for the comments that used negative words to convey a positive idea and vice versa.

## Results

### Analysis of the Metadata

The metadata reported are for the four promotional materials used in the campaign, which included a video and three banner advertisements. The total number of likes, shares, and views were obtained from the promotional materials’ respective Facebook pages. Table 2 presents this information along with the number of comments for each posting. The video had 203,754 views and together the promotional materials generated 819 comments, 6125 likes, and 300 shares.

### Thematic Analysis

After several iterations over the number of unsupervised classification themes extracted, the best theme clarity was obtained for the singular value decomposition that extracted four themes. By using the cut-off weights, the keywords and archetypes for the themes were established. The comments above the cut-off weight formed the archetypes and the numbers for each of the theme are shown in Table 3. The archetype comments were reviewed and the four themes were labeled as risk of pregnancy, alcohol and culture, credibility of the campaign, and contraception failure (Table 3).

**Table 2:** Metadata for the campaign posting.

Posting	Number of comments	Likes	Shares	Views
Don’t Know? Don’t Drink (video)	146	1281	92	203,754
Missed a pill? Maybe? (ad #1)	330	2127	101	N/A^a^
You used a condom, right? (ad #2)	250	1714	69	N/A
Definitely, definitely not pregnant? (ad #3)	86	1003	38	N/A
Don't Know? Don't Drink profile thumbnail	7	0	0	N/A
Total	819	6125	300	203,754

^a^N/A: not applicable.

**Table 3:** Themes and their corresponding keywords.

Labels	Term cut-off	Keywords	Comment cut-off	Number of archetypes^a^	Exemplar^b^
Risk of pregnancy	0.317	sex, pregnancy, woman, mean, contraception	0.318	67	“...No contraception is 100%. What a dumb ad”
Alcohol and culture	0.320	wine, eat, raw, fish, pregnancy,	0.331	69	“Explain all the European pregnant women who drink wine on a daily basis because it’s a cultural habit...?”
Credibility of the campaign	0.288	stop, stupid, risk, drink, problem	0.278	49	“What a stupid campaign, everything has ‘risks’ but this just seems like a big fat waste of time.”
Contraceptionfailure	0.319	pill, miss, condom, time, period	0.352	35	“...The ad should be ‘missed a pill? wear a condom’(or probably get him to wear one hahaha)”

^a^The number of comments with meaning-making theme information weight above a critical level.

^b^Exemplars are snippets of the archetypes with the highest information weight for that meaning-making theme.

### Logistic Regression Analysis

Using logistic regression, we investigated whether the contents of the message were related to, or predicted by, the comments. The stepwise regression retained coefficient estimates for two of the themes, namely, “risk of pregnancy” and “alcohol and culture” using the critical alpha value of .05 (Table 4). The −2log-likelihood ratio statistics showed that adding the two themes improved the model substantially (Intercept only=196.233; intercept and covariates=157.873). The chi-square test statistics for adding the two themes into the model (Risk of pregnancy: Wald χ^2^=8.7591, *P*=.003; culture and drinking: Wald χ^2^=18.5995, *P* ≤.001) were supported with evidence at the critical alpha level (Table 4), confirming their inclusion in the model was supported.

The baseline odds for strong evidence in the comments that the message was received and reflected in the themes was one in three (0.33). The odds of this message reflection approximately doubled when the first meaning-making theme (risk of pregnancy) was included (see Table 4). When evidence for the second meaning-making theme (alcohol and culture) was included, the odds increased by about three times (see Table 4). Based on the odds ratio, the probability of finding evidence for the message being received and reflected in the two themes is shown in Table 5. The other themes showed no strong evidence of association with the supervised classification message reflection theme. The authors inspected the exemplars for these other two themes and were satisfied that the meaning-making was not related to the message but rather to unrelated topics.

### Sentiment Analysis of the Themes in the Model

Sentiment analysis was conducted to further illuminate the interpretation of the regression model results. Using Mathematica [31], qualifying comments containing strong evidence of the two meaning-making themes in the model (“risk of pregnancy” and “alcohol and culture”) were categorised as positive, neutral, and negative. The validity of this categorization was then checked in the qualifying comments by the authors and found to be satisfactory. Table 6 presents the results of the sentiment analysis. As the results show, this campaign evoked all three sentiment valences: positive, neutral, and negative. For both the unsupervised classification meaning-making themes extracted, the proportions of negative comments were higher than the positive and neutral comments (Table 6).

**Table 4:** Association between the meaning-making themes and message.

Parameter	Degrees of freedom	Estimate	Standard error	Wald χ^2^	*Pr*>χ^2^	Exp (Est)
Intercept	1	−1.1058	0.2693	16.86	<.001	0.331
Risk of pregnancy	1	0.7782	0.2629	8.76	.003	2.177
Alcohol and culture	1	1.1161	0.2588	18.60	<.001	3.053

**Table 5:** Probability of the message provoking a message-reflective response.

Parameter	Probability
Without any meaning-making	0.25 (0.331/1+0.331)
Risk of pregnancy cognition	0.42 (0.331×2.177/1+0.331×2.177)
Alcohol and culture cognition	0.50 (0.331×3.053)/1+(0.331×3.053)
All related cognition	0.69 (0.332×2.177×3.053)/1+(0.332×2.177×3.053)

**Table 6:** Sentiment analysis of comments in the two themes.

Meaning making themes	Positive comments n (%)	Neutral comments n (%)	Negative comments n (%)
Risk of pregnancy	15 (23)	18 (27)	33 (50)
Alcohol and culture	23 (34)	15 (22)	30 (44)
Total	38 (28)	33 (25)	63 (47)

## Discussion

### Principal Findings

Despite the lack of empirical evidence, observed in the literature, the use of Facebook for communicating health messages is gaining popularity [19]. The RTCs that attempted to develop social media as vehicles for health promotion have tested and reported favorable results [20-23]. All the same, these studies were on specific features of Facebook, such as the messaging system, and the authors implicitly advocated Facebook as a platform for health promotion. The findings of such studies have provided the impetus for public health researchers and professionals to use Facebook and other social media as a format for delivering health promotion messages. This study aimed to investigate the use of Facebook’s advertising channel for health promotion to generate hypotheses for the systematic development of this platform. The findings of this study confirm high levels of engagement, as observed in previous research [25]. When examining the level of engagement, the findings were based on the metadata Facebook provided for the campaign. The overall number of likes (6125), shares (300), and comments (819) achieved in a short time (June to September 2015) suggests the promotional material stimulated reasonable engagement within the target audience. The evaluative worth of the metadata is hard to establish. All the same, the high level of engagement suggests that this channel was successful, both in presenting materials to a target audience and for facilitating engagement. This observation confirmed the earlier reporting of Platt et al [25], suggesting that this channel encouraged user engagement.

The video used in this campaign produced an impressive number of views (203,754). However, it is hard to reach a conclusion on how the message was received based on this metadata alone. Going by the definition of likes, the number of people who approved the video was less than one percent (0.63%), that is, 1281 likes given by 203,754 individuals who viewed the video (see Table 2). The reaction of the remaining 99% remains unknown. The same can be claimed for shares, which was 0.05%, that is, the proportion of the number of shares from those who viewed the video (see Table 2). Whereas sharing expanded the campaign’s reach to other audiences, the context in which they were shared remained unknown. As such, whether the message was supported or rejected could not be ascertained based on the measures of engagement observed in this and a previous study [25].

This study goes further than the previously reported study [25] to investigate the association between the meaning-making themes generated by the comments and the reflected message in the themes. The exemplars in Table 3 suggest that the comments seemingly dismissed the campaign (the carrier of the message) as “a big waste of time” (see exemplar for credibility of the campaign in Table 3) and as a “dumb ad” (see exemplar for risk of pregnancy in Table 3). Consequent to such dismissal, the likelihood of the message being communicated “as intended” is small. Falomir & Invernizzi [32] observed similar reactions to antismoking messages. A key factor responsible for the dismissal was the “smoker identity,” reenforced by the social categorization of individuals into smokers and nonsmokers. When such caegorization becomes a defining trait of oneself, people tend to be defensive of their self-identity [33,34]. Like smoking, drinking alcohol is common practice for many people and hence, may contribute to defining their self-identity.

The findings from the logistic regression indicated the base level likelihood of the message being featured in the comments was comparatively small (1 in 3; see in the column Exp [Est] for the intercept in Table 4). The odds increased by a combined factor of about six when the two themes were included in the equation (odds ratio of 2.177 for risk of pregnancy; odds ratio of 3.053 for alcohol and culture). The overall probability of the message receiving any reaction was about seven in ten (0.69, Table 5). The sentiment analysis performed for the two themes returned neutral, positive, and negative, with the latter, unfortunately for the sponsoring stakeholder and the public health of the target population, trending higher (47%) than the former two (28% for positive and 25% for neutral comments, Table 6). As discussed above, if self-identity is a contributing factor for drinkers to react negatively to the message of abstinence if they are unsure of pregnancy, then it is even more likely that the message will not receive the endorsement of those giving negative comments. It is possible that the overly negative reaction from women for the Don’t Know? Don’t Drink campaign may well be in defense of their self-identity. However, as evidence to support this is currently lacking, future research to test the self-identity theory for women who drink alcohol is warranted (H1).

Negative comments can influence subsequent viewers’ evaluations, as Walther et al [35] observed for antimarijuana messages disseminated via YouTube. Walther and colleagues found that the messages with negative comments were perceived as less effective than those with positive. Shi et al [36] reported a similar pattern in the evaluation of antitobacco messages, showing how negative comments dissuaded effectiveness. Identification with the previous commenter influenced the direction of the perceived effectiveness [35,36], as explained by Conformity Theory [37]. According to this theory, people conform to the behavior of their peers to gain social approval. In a virtual context, conformity to an identity is conveyed via commenting. With a large number of the target population identifying themselves as drinkers (75%) [38], the accumulation of negative comments directed at this campaign could progressively render it ineffective. Whether or not peer pressure to conform was the underlying cause for the large proportion of negative comments observed in this study, needs further investigation (H2). It could be argued that the individuals whose comments were neutral could swing either way. However, it is highly likely that peers providing negative comments loaded with anecdotal evidence can convince the neutral commenters who identify themselves as “drinkers” to reject the message, albeit this premise needs to be investigated (H3).

Sentiment analysis was carried out for the two themes included in the logistic regression equation. The proportion of positive comments (28%— total for risk of pregnancy and alcohol and culture in Table 6) was small compared with the negative ones (47%—total for risk of pregnancy and alcohol and culture in Table 6). Whether the women who made positive comments knew the dangers of drinking during pregnancy or whether their position was modified by the campaign can only be confirmed using a cross-sectional survey design. Therefore, a future study is required to investigate whether the campaign produced attitudinal change among those who gave positive comments (H4). Nevertheless, their presence in the current data suggests the campaign reached a broad cross-section of women.

The Don’t Know? Don’t Drink campaign adopted a one-way communication format. That is, the message, encoded in promotional materials, was disseminated in one direction via Facebook’s advertising channel. The line of communication was unidirectional with no further interaction between the sender and the receivers. Evaluation of such social campaigns is carried out separately, usually by an independent agency. In the current instance, the comments provided are a first-hand assessment of the acceptability of the message. The findings from the logistic regression and the sentiment analysis revealed that the message was mostly “not” received “as intended” by those most vulnerable to an alcohol-exposed pregnancy. This study cautions the use of a one-way communication model for conveying such warning messages via Facebook’s advertising channel. At most, it could be said that the communication process was initiated. The communication effectiveness could be enhanced using a two-way communication format, which enables the promoter to respond to the negative comments. Thus, the negative comments could be seen as opportunities, both to provide scientific evidence and reinforce the message. To understand the communication process further, the testing of Facebook’s advertising channel using a two-way communication format is warranted (H5).

Facebook offers the option to moderate the comments by hiding or deleting them to prevent derogatory comments being viewed by others. The temptation to moderate the negative comments by managers of such public campaign needs to be recognized as it can impact negatively on the findings. However, in this study, as the number of negative comments was greater than the positive ones for the campaign, it is very unlikely that this was the case.

In a two-way communication, such negative comments open the channel of communication to engage back with the commenters with additional explanation and information. In doing so, concrete evidence is placed in the domain of the target audience. Hence, negative comments must be viewed as a means to an end, which is to make the current information available to the target population, thereby alleviating the knowledge gaps. Therefore, we suggest that public campaigns using social media include a protocol against moderating the comments solely based on sentiment valence so as to maximize the intervening opportunities and maintain the completeness and quality of the raw data.

### Conclusions

The user engagement observed in this study was consistent with previous research. The examination of the comments revealed that the message was not received favorably by a majority of women. The observations made in this study provided a direction for future research, to both understand the target audience (traits of women who drink alcohol) and enhance the effective use of Facebook’s advertising channel for health promotion activities. Future investigations are required to find out whether the self-identity and conformity theories can explain why the abstinence in a pregnancy message was largely unaccepted. Although the positive comments were fewer in number compared with the negative, they also need to be studied to find out whether that attitude resulted from the campaign or they preexisted. The negative comments should be seen as an opportunity for engagement with the target population.

The one-way communication format used in the current campaign could only observe the commencement of the communication process. Future investigation is needed to find out whether a two-way communication format would help both promoters and researchers to engage back, which might improve the acceptability of public health messages delivered via social media. Finally, the observations made in this study caution against using a one-way communication format in conjunction with Facebook advertising to convey warning messages.
